# Implications of Self-Other Overlap for Cyber Dating Abuse in Young Adult Romantic Partners

**DOI:** 10.3390/bs14111037

**Published:** 2024-11-04

**Authors:** Miriam Parise, Silvia Donato, Ariela Francesca Pagani

**Affiliations:** 1 Department of Psychology, Family Studies and Research University Centre, Università Cattolica del Sacro Cuore, Milano Largo Gemelli, 1, 20123 Milano, Italy; miriam.parise@unicatt.it (M.P.); silvia.donato@unicatt.it (S.D.); 2 Department of Humanities, University of Urbino Carlo Bo, Via Saffi, 15, 61029 Urbino, Italy

**Keywords:** self-other overlap, cyber dating abuse, social network sites, young adults, romantic relationships

## Abstract

Social network sites (SNSs) have brought about profound changes in the way people relate to others, including their romantic partners. Despite the advantages SNSs may have for building and managing romantic relationships, their use can be linked to risky behaviors within romantic relationships, such as the emergence of jealousy, control, and intrusiveness, i.e., cyber dating abuse (CDA) behaviors. The present study, in a sample of 315 Italian young adults involved in a romantic relationship (74.6 percent women and 25.4 percent men) aged 20 to 33 years (*M* = 24.17; *SD* = 2.60), explored CDA behaviors and their association with self-other overlap. Findings showed a positive association between self-other overlap and the frequency of CDA behaviors. That is, those who struggled to recognize their partners as different from themselves tended to control and enact intrusive behaviors toward them. This association, however, was moderated by the partners’ relationship duration, so that it was only significant for partners in a long-term relationship. The study expands our understanding of CDA behaviors in romantic relationships, contributing to identifying the conditions under which they are more likely to be perpetrated. In addition, it helps inform interventions for preventing risky behaviors within young adults’ romantic relationships.

## 1. Introduction

### 1.1. Cyber Dating Abuse as an Emerging Risky Behavior Among Romantic Partners

Social Network Sites (SNSs) have become an essential tool for contemporary social interaction [1]. As of January 2024, global statistics indicate that 62.3% of the world’s population is active on these platforms and that this percentage is constantly increasing [2]. In Western countries, such as Italy, this percentage reaches almost 80% of the population over the age of 18 [3]. The significant success of SNSs can be mainly attributed to the role they play in facilitating and maintaining interpersonal relationships with one’s extended social context [4] and in satisfying basic social and psychological needs, such as self-presentation, sense of belonging, self-disclosure, social support, and intimacy [5].

The impact of SNSs is also demonstrated in the context of romantic relationships [6]. SNSs provide additional opportunities for communicating and interacting with the partner and increase feelings of connection ([7,8,9,10], as they enable the overcoming of physical distances through the immediate exchange of textual and multimedia content [11]. However, SNSs may provide the context for potentially risky behaviors toward the partner [12], especially among adolescents and young adults for whom digital technology is an integral part of daily life [13]. In fact, research has documented multiple abusive behaviors among partners through digital interaction that can be described as forms of cyber dating abuse (for an extensive review see [14,15,16]). Cyber dating abuse (CDA) behaviors consist of “using mobile phones and digital social networks to control the partner, limit his/her freedom, mock, denigrate, threaten, and/or force him/her to perform or suffer unwanted sexual acts” ([17], p. 2). This phenomenon is a manifestation of Intimate Partner Violence [14] and can occur both in stable and sporadic relationships e.g., [18].

### 1.2. Variability in Cyberdating Abuse Behaviors

CDA has been a constantly growing phenomenon among young people using digital technology [14]. However, some variability linked to socio-demographic characteristics seems to exist. In particular, some studies highlight gender differences in CDA behaviors, although results are not univocal, with studies showing males to have a higher probability of both perpetrating and being victims of CDA practices, e.g., [19,20], while other studies indicate the opposite, e.g., [21,22]. In addition, some research evidence points to an absence of significant differences between genders, e.g., [23,24,25]. Research also highlights some differences related to age, but again, results are not consistent. According to some studies, being older is associated with a higher probability of perpetrating CDA [20,26]. However, according to other studies, the youngest seem to be more at risk of experiencing CDA [23], while other studies do not find differences related to age [15]. Relationship duration appears to be another important factor modulating CDA. Although some evidence demonstrates the absence of differences [27], other studies found that people in longer relationships enacted more CDA behaviors [19].

### 1.3. Predictors of Cyberdating Abuse Behaviors

In recent years, the magnitude of CDA in terms of the effects on the partners’ safety and mental health has been documented [23,28,29]. In fact, the literature underlines that both CDA perpetrators and victims are characterized by maladjustment and lower levels of well-being [30,31]. Therefore, it appears particularly important to deepen our knowledge of the potential predictors of cyber dating abuse in order to prevent it. Recent systematic reviews or meta-analyses demonstrated that CDA perpetration could be predicted by individual, relational, and sociocultural variables [15,16]. Individual variables are related, for example, to anger/hostility, psychopathy, narcissism, vulnerability, and grandiosity. Relational variables may refer to the legitimization of CDA, jealousy, attachment, and experiences in the family. Concerning sociocultural variables, research has identified beliefs in myths about love, sexist beliefs, and gender stereotypes as precursors of CDA.

Despite the growing interest in cyber dating abuse, the research on the predictors of its perpetration still needs to be expanded [32]. In particular, scarce attention has been given to potential predictors related to identity dimensions. As a matter of fact, although the psycho-social literature is unanimous in recognizing identity aspects as powerful motivators of behaviors, e.g., [33], surprisingly, research on CDA is lacking in studies exploring this issue. In particular, behaviors and dynamics occurring within interpersonal relationships are guided by relational aspects of the self (i.e., how people represent themselves in relation to their partner), e.g., [34,35,36]. Although research has focused mostly on the effects of the relational self on offline relational behaviors, it is reasonable to expect that the representation of the self-in-relationship could also have consequences on online behaviors towards one’s partner.

### 1.4. Self-Other Overlap in Romantic Relationships

As individuals develop a close relationship, they progressively restructure their identities. Their representation of themselves changes and they come to perceive themselves less as single individuals (“Me”) and more as part of a relational unit, i.e., the couple (“We”) [34,36]. This phenomenon has been described by different concepts such as cognitive interdependence [34], couple identity [37,38], and inclusion of the other in the self [39]. Although underlining slightly different nuances, these concepts share the idea that, within relationships, the boundaries between the partners’ selves change and the cognitive representations of themselves tend to merge. That is, couple members tend to integrate the resources, perspectives, and characteristics of their partner in the relationship and include them in their self-concept, leading to the creation of a kind of self-other overlap [39,40].

Self-other overlap is generally considered a desirable feature of relationships. In fact, greater overlap is linked to greater closeness, intimacy, commitment, relationship quality, and stability [40]. In addition, self-other overlap promotes pro-relationship behaviors by motivating the use of behaviors that serve to protect and sustain ongoing relationships, e.g., [41,42]. However, recent evidence points to possible negative behavioral repercussions of self-other overlap [43]. In particular, self-other overlap was associated with destructive conflict behaviors in the couple relationship [44]. Moreover, in cultures of honor, self-other overlap predicted intimate partner violence [45]. Following this perspective, a higher overlap may lead to an inability to recognize the boundaries between oneself and the partner that, in turn, may lead to disrespecting the other [46,47]. Therefore, it can be that when identity boundaries are too blurred, individuals struggle to distinguish one’s thoughts and emotions from their partner’s and may be more prone to enact intrusive/controlling and even abusive behaviors towards the other.

### 1.5. The Current Study

As outlined above, how people represent the boundaries between oneself and the other has repercussions on offline relational behaviors. Although these results are to some extent inconsistent, they suggest that self-other overlap may modulate how partners behave in online contexts as well. However, the implications of self-other overlap for online behaviors have been neglected by researchers. As a matter of fact, both the literature focusing on CDA and the literature on relational identity and self-other overlap has not taken into consideration the link between these two variables.

The current study aims to begin filling this gap by focusing on the potential link between self-other overlap and CDA perpetration. Addressing this lacuna is important for several reasons. First, exploring the role of identity variables for CDA behaviors may help enrich our understanding of a growing risky phenomenon like CDA. In addition, the study of the relationship between CDA and self-other overlap could lead to an advancement and integration of the literature on CDA and the literature on self-other overlap. Finally, a deeper understanding of the conditions in which cyber dating abuse behaviors may occur can be a first step towards its prevention.

Specifically, we formulated two research questions as follows:

- (RQ1) Is self-other overlap associated with the perpetration of cyberdating abuse behaviors? In line with the studies highlighting a positive effect of self-other overlap and relational behaviors [41,42], we could expect that self-other overlap, by promoting a sense of closeness and mutual understanding between partners together with a pro-relationship attitude, may help partners to not enact cyber dating abuse behaviors. If partners perceive a commonality in their resources, perspectives, and characteristics, they may not feel the urge to intrude or control the other. However, alternatively, it could also be that the blurring of boundaries between oneself and the other may lead to a scarce recognition of the partner’s inherent difference and in turn lead to behaviors that are disrespectful or abusive towards them [46,47].
- (RQ2) Is the relationship between self-other overlap and the perpetration of cyberdating abuse behaviors moderated by socio-demographic variables? In this study, in line with the literature, to check for potential variability in CDA perpetration, we explored the moderating role of gender, age, and relationship duration. Again, given the absence of univocal evidence in the literature on the effects of these socio-demographic variables, we did not formulate specific hypotheses. In fact, although some studies did not document the effects of gender, age, and relationship duration in processes related to CDA behaviors [15,17,23], other studies detected some variability related to these socio-demographic characteristics, although not in univocal directions, e.g., [19,20,21,23].

## 2. Materials and Methods

### 2.1. Participants and Procedure

Participants were recruited via the snowballing method. In particular, we used advertisements and posts on social network sites like Facebook, Instagram, and X. To participate, individuals had to meet the following inclusion criteria: (1) being between 20 and 35 years old, and (2) being involved in a romantic relationship. Three hundred and fifteen young adults participated in the research: 74.6% were women (N = 235) and 25.4% were men (N = 80). The age range of individuals was 20–33 years (*M* = 24.17, *SD* = 2.60) and 98.7% of them declared to be Italian. At the time of the study, participants had been in a romantic relationship for an average of 3.33 years (*SD* = 2.72); 94.9% of them were unmarried, 4.8% were married, and 0.3% were separated or divorced. Moreover, 17.3% of the participants lived with their partner. Most individuals had no children (97.1%), although 1.6% of couples had one child, and 1.3% had two children. Concerning sexual orientation, 93.7% of the sample declared to be heterosexual, 4.8% bisexual, 1.3% homosexual, and 0.3% pansexual. The sample was composed of 32.4% students, 30.5% of workers, and 32.4% student-workers. Among those who worked, 48.5% had a full-time job, while 51.5% had a part-time job.

Participants completed an online self-report questionnaire lasting approximately 30 min through the Qualtrics platform. The questionnaire assessed socio-demographic variables, habits related to SNSs, and aspects of relational functioning. All individuals took part voluntarily in the research and provided informed consent prior to the beginning of the study. To ensure anonymity, the data collected through the Qualtrics platform were password protected. When the data were downloaded for data analysis, they were stored in a password-protected personal area of the team researchers’ computer, as required by the Regulation (EU) 2016/679 of the European Parliament. The study was conducted in accordance with the Declaration of Helsinki and approved by the Ethical Committee of the Università Cattolica del Sacro Cuore, Milan (Ref. 49/24).

### 2.2. Instruments

#### 2.2.1. Socio-Demographic Characteristics

Participants firstly completed items on socio-demographic variables among which were gender, age, and relationship duration.

#### 2.2.2. Self-Other Overlap

To assess self-other overlap, we used the Inclusion of the Other in Self scale (IOS) [48], which is a pictorial measure composed of 7 Venn-like diagrams, each of which is composed of 2 circles varying in their degree of overlap. Participants were asked to select the diagram that best represents their relationship with their partner.

#### 2.2.3. Cyber Dating Abuse Perpetration

To assess cyber dating abuse, we used the Cyberdating Q_A scale [27]. This scale is composed of 28 items rated on a 7-point scale (from 1 = never to 7 = always) tapping different cyber dating behaviors that individuals can enact towards the partner. In particular, it is composed of six subscales as follows: “online control” (item example: “I have opened a fake account so that my partner adds me and I can control him/her”), “emotional communication strategies” (item example: “I change the way I write when I want my partner to ask me what is wrong”), “online jealousy” (item example: “I get jealous when my partner posts provocative photos on their social network profile”), “online intrusive behavior” (item example: “When I’m annoyed and my partner does not respond, I leave a lot of messages on their social network site”), “cyber dating practices” (item example: “I have ‘flirted’ with other people via social media whilst in a relationship”), and “online intimacy” (item example: “I have a really good time with my partner when we’re online together”). For this project, we focused on online negative behaviors towards the partner by computing a composite index of the subscales “online control”, “online jealousy”, and “online intrusive behavior”, in order to have a measure of cyber dating abuse perpetration. Cronbach’s alpha for the index was equal to 0.81.

### 2.3. Data Analyses

Before testing our core predictions, preliminary analyses were conducted to describe the sample in terms of the levels and associations among the variables of interest. In particular, we performed descriptive analyses, independent sample *t*-tests, and linear correlations (Pearson’s *r*) using the SPSS software (v. 28). Then, multiple linear regression was used to examine the association between self-other overlap and cyber dating abuse and the potential moderating role of gender, age, and relationship duration in this association. Specifically, we conducted three multiple linear regressions, one for each moderating variable, using the SPSS software (v. 28). In these regressions, cyber dating abuse was entered as the dependent variable, while self-other overlap, one moderator (gender, age, or length of relationship), and the interaction term between self-other overlap were entered as predictors. Whenever a significant interaction effect emerged, simple slope tests were conducted to explore how the relationship between self-other overlap and cyber dating abuse behaviors changed according to different levels of gender, age, and relationship duration. Simple slope testing was conducted using the Interaction! software [49].

## 3. Results

### 3.1. Preliminary Analyses

As shown in Table 1, participants reported medium-high scores of self-other overlap (*M* = 5.18; *SD* = 1.29) and relatively low levels of cyber dating abuse behaviors (*M* = 1.93; *SD* = 0.83). Moreover, independent sample *t*-tests showed that men (*M* = 5.45; *SD* = 1.10) reported significantly higher [*t* (313) = 2.36, *p* = 0.03] levels of self-other overlap than women (*M* = 5.09; *SD* = 1.34), while no gender differences emerged in terms of cyber dating abuse behaviors [*t* (313) = −1.29, *p* = 0.70; men: *M* = 1.82; *SD* = 0.76; women: *M* = 1.97; *SD* = 0.85]. Finally, linear correlations showed a significant positive association between self-other overlap and cyber dating abuse behaviors (*r* = 0.119, *p* = 0.035) and a significant negative association between cyber dating abuse behaviors and age (*r* = −0.119, *p* = 0.035). No other significant associations emerged.

### 3.2. Primary Analyses: Association Between Self-Other Overlap and Cyber Dating Abuse Perpetration and the Moderating Role of Gender, Age, and Relationship Duration

**Figure 1:**
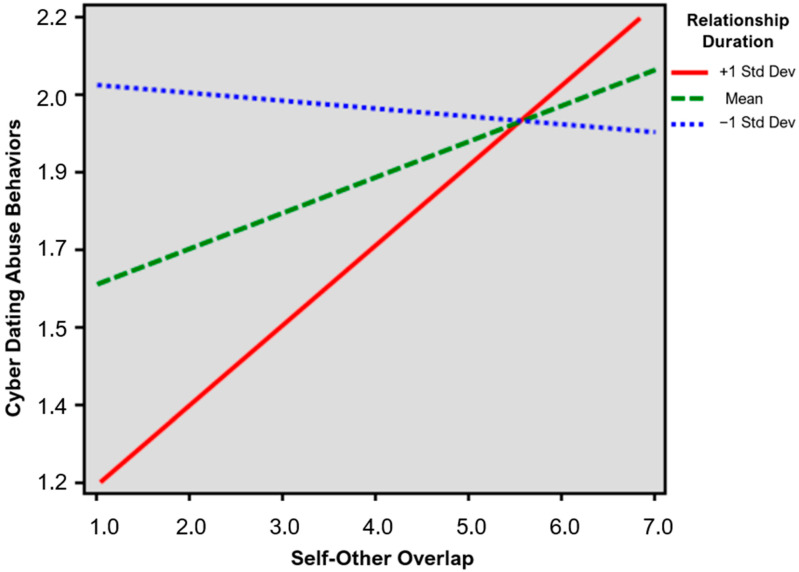
Moderating role of the relationship duration in the association between self-other overlap and cyber dating abuse behaviors.

The results of the regression in which gender was included as a moderator showed a significant and positive association between self-other overlap and cyber dating abuse behaviors (b = 0.084, *t* = 2.11, *p* = 0.035); the more individuals perceived an overlap between the self and the partner, the more they engaged in cyber dating abuse behaviors towards their partner. No other associations were found. The Adjusted R^2^ of the model was 0.012. Again, the results of the regression in which age was included as a moderator showed only a significant and positive association between self-other overlap and cyber dating abuse behaviors (b = 0.069, *t* = 1.935, *p* = 0.053). The Adjusted R^2^ of the model was 0.018. Instead, the results of the regression in which the duration of the relationship was included as a moderator showed, in addition to a significant and positive association between self-other overlap and cyber dating abuse behaviors (b = 0.074, *t* = 2.074, *p* = 0.038), a significant and negative association between the duration of the relationship and cyber dating abuse behaviors (b = −0.015, *t* = −2.959, *p* = 0.003). This means that cyber dating abuse behaviors towards the partner decreased as the duration of the relationship increased. In addition, a significant and positive interaction between self-other overlap and relationship duration (b = 0.002, *t* = −2.864, *p* = 0.004) emerged. The Adjusted R^2^ of the model was 0.032. The simple slope analysis (Figure 1) showed that the association between self-other overlap and cyber dating abuse behaviors was stronger for those in a relationship of a longer duration (+1 *SD*, b = 0.165, *t* = 3.502, *p* < 0.001), whereas this association was no longer significant for those in a shorter relationship (−1 *SD*, b = −0.016, *t* = −0.342, *p* = 0.366).

## 4. Discussion

At present, virtual interactions through SNSs are increasingly used by young people as a way of managing their romantic relationships, and within this context, risky online behaviors may occur [50]. Cyber dating abuse is one such behavior and consists of using digital social networks to control the partner, limit their freedom, mock, denigrate, threaten, and/or force them to perform or suffer unwanted sexual acts [17]. Given that cyber dating abuse can have serious consequences for the youth, it is important to better understand it to develop more effective prevention strategies. In particular, the effort of researchers has been increasingly directed towards the identification of those factors that can predict the perpetration of CDA behaviors.

The present study moved towards this direction and explored the relationship between cyber dating abuse and self-other overlap in a sample of young adults in a romantic relationship. Self-other overlap reflects the degree to which individuals in a romantic relationship, through a process of inclusion of the other in the self, come to progressively restructure the cognitive boundaries of themselves to incorporate their partners in the cognitive representation of themselves [39,40]. Although it has never been studied in relation to cyber dating abuse, self-other overlap may play a critical role in its enactment. In fact, how people represent the other in relation to oneself may guide behaviors towards them [34].

### 4.1. The Association Between Self-Other Overlap and the Perpetration of Cyber Dating Abuse Behaviors

With regard to RQ1, in all our regression analyses, self-other overlap was positively associated with the perpetration of cyber dating abuse behaviors. That is, the more individuals perceived their partners as overlapping to themselves the more they were prone to display jealous, controlling, or intrusive online behaviors towards them. The literature on the process of inclusion of the other in the self has often emphasized its positive effects on couples, showing for instance that the more the partners perceive themselves as overlapping the more they have happy and long-lasting relationships and the more they are able to enact pro-relationship behaviors [40,43]. According to this perspective, a higher level of inclusion of the other in the self may facilitate a deeper knowledge of the partner and therefore reduce the need to control him/her. In addition, a higher inclusion of the partner in the self may lead to consider the partner as more similar to oneself and to treat them as one would want to be treated, thereby refraining from enacting those behaviors that may appear as an intrusion. The present results, however, in line with some recent studies [44,45], seem to underline another facet of the process of inclusion of the other in the self. It is likely that when individuals perceive the other as overlapping, they are less able to perceive the differences and the boundaries between oneself and the partner and in turn feel more entitled to not respect such boundaries. In addition, feeling like a single entity with one’s partner may lead individuals to tolerate less that the other might have individual or unshared aspects of life and thus feel the urge to control them. This is consistent with the previous literature showing that difficulties in the recognition of the difference between oneself and the partner are linked to higher levels of intrusiveness and control [46,47].

### 4.2. The Moderating Role of Gender, Age, and Relationship Duration

With regard to RQ2, regression analyses did not highlight any effect related to gender and age, in line with some studies that did not find any gender or age differences in CDA, e.g., [23,25]. That is, the intensity and direction of the association between self-other overlap and CDA perpetration did not differ between men and women nor between younger and older participants. In this regard, it must be noted that our sample was composed mainly of females and that, despite a good age variability, the vast majority of participants were between 21 and 25 years old, which may have had some influence on the results obtained. Instead, we found effects related to relationship duration. In particular, differently from existing evidence [19], we found a direct effect of relationship duration on CDA, with cyber dating abuse behaviors decreasing as the duration of the relationship increased. However, moderation analyses were to some extent more in line with the literature [19], showing that the association between self-other overlap and cyber dating abuse behaviors was significant for those with a longer relationship duration only. It could be that young adults who have invested time and energy in building a sense of unity with their partner are more anxious about losing their relationship and their partner. For these individuals, the enactment of cyber dating abuse behaviors could then be an attempt to maintain a sense of control over the partner and preserve the relationship from possible threats. It may also be that, in more established relationships (more than in shorter ones), when overlap is not mitigated by the recognition of the partner’s inherent differences as well as their uniqueness, it can be particularly associated with potentially disrespectful behaviors towards them.

### 4.3. Limitations and Future Research

The present study should be considered in light of the following limitations. First, due to the cross-sectional and correlational design of the study, conclusions about causality and the direction of effects cannot be drawn. Future research could benefit from using longitudinal data to confirm the direction of the effects. In addition, our sample was composed mostly of women. Although in our findings gender did not emerge as a predictor or a moderating variable, it would be important to collect data from gender-balanced samples to test for possible gender differences in the patterns found. Moreover, it must be noted that the effect sizes were weak, and future replications are needed to confirm these results. Also, our analyses explored the moderating role of socio-demographic variables like gender, age, and relationship duration in the association between self-other overlap and CDA perpetration. We selected these variables to bring our research into dialogue with the existing literature, but we are aware that other variables, both socio-demographic, such as sexual orientation, and those related to psychological and relational functioning, such as personality traits and relationship satisfaction, could modulate this process. Finally, our measures assessed individual self-perceptions collected from one informant. The adoption of a dyadic perspective and the involvement of both members of the couple could be useful for better investigating relational dynamics between partners.

## 5. Conclusions

This study sheds light on cyber dating abuse behaviors in romantic relationships, helping identify the conditions under which they are more likely to be perpetrated. The study has implications for both research and practice.

As for research implications, it expands and integrates two distinct literatures. First, it expands the literature on CDA predictors, exploring an under-investigated issue such as the role of self-other overlap in the perpetration of CDA behaviors. Results point out that the way in which young adults represent themselves with respect to their partner plays a role in the enactment of cyber dating abuse behaviors, especially for those in a long-term relationship. Moreover, our findings expand the literature on self-other overlap. Although self-other overlap can be beneficial for romantic partners, it may also have some downsides and, in some circumstances, expose the relationship to potential negative consequences.

As for practical implications, our study highlights relevant aspects to be targeted by interventions aiming at preventing risky behaviors in romantic relationships and promoting young couples’ well-being. First, these interventions should incorporate modules on online behaviors, given their growing role in the management of romantic relationships. Covering these topics is useful in increasing partners’ awareness of how the relationship is shaped through both offline and online interactions. In addition, couples should be invited to reflect on issues related to relational identity, such as similarities, differences, and boundaries between partners. Considering how the representation of the self and the partner is critical for the enactment of behaviors could help prevent risky behaviors in the relationship. This can be especially important in the case of online behaviors, which expand partners’ potential for connection, but also for intrusiveness. Finally, these topics should be addressed from the early stages of the relationship, in an attempt to prevent the development of maladaptive self-representations and behaviors in the subsequent phases of the relationship. More broadly, in line with the Sustainable Development Goals (SDGs) indicated by the United Nations, targeting these issues may contribute to fostering healthier, non-violent relationships, thereby promoting good health and well-being and reducing inequalities.

## Figures and Tables

**Table 1:** Descriptive statistics and correlation among variables of the study.

	M	SD	1	2	3	4
1. Self-other overlap	5.18	1.29	-			
2. Cyber dating abuse behaviors	1.93	0.83	0.119 *	-		
3. Age	24.17	2.60	−0.042	−0.119 *	-	
4. Relationship duration	3.33	2.72	0.022	−0.039	0.367 ***	-

Notes. *** *p* < 0.001, * *p* < 0.05.

## Data Availability

The raw data supporting the conclusions of this article will be made available by the authors on request.
